# Occurrence and clinical course of immune checkpoint inhibitor-associated inflammatory arthritis and polymyalgia rheumatica-like syndromes: a systematic review and meta-analysis

**DOI:** 10.3389/fimmu.2026.1959233

**Published:** 2026-09-30

**Authors:** Chenyang Li, Ruhui Fu, Xinxin Geng, Yunru Zhang, Qiang Yuan, Peifeng Li, Yanan Fan, Ying Zhang

**Affiliations:** 1 Luoyang Orthopedic-Traumatological Hospital of Henan Province (Henan Provincial Orthopedic Hospital), Luoyang, China; 2 Fujian University of Traditional Chinese Medicine, Fuzhou, Fujian, China; 3 Henan University of Traditional Chinese Medicine, Zhengzhou, Henan, China

**Keywords:** immune checkpoint inhibitor, inflammatory arthritis, meta-analysis, polymyalgia rheumatica, real-world evidence, rheumatic immune-related adverse event, SWiM, systematic review

## Abstract

**Background:**

Immune checkpoint inhibitor-associated inflammatory arthritis (ICI-IA) and polymyalgia rheumatica (PMR)-like syndromes are clinically important rheumatic immune-related adverse events, but definitions vary across studies. We estimated their occurrence in real-world cohorts and summarized clinical course and management.

**Methods:**

PubMed, Ovid MEDLINE, Embase, Web of Science Core Collection, and Cochrane CENTRAL were searched from inception through July 25, 2026. Two reviewers independently screened studies, extracted data, and assessed risk of bias, with disagreements resolved by a third reviewer. Compatible single-group proportions were pooled using random-intercept binomial generalized linear mixed models; non-poolable clinical-course evidence was synthesized using the Synthesis Without Meta-analysis framework.

**Results:**

Sixty-five reports representing 63 studies were included. Five independent ICI-treated cohorts reported 140 study-defined *de novo* ICI-IA cases among 4,644 patients, yielding a pooled crude occurrence proportion of 2.87% (95% CI, 2.02%-4.07%; 95% prediction interval, 1.06%-7.53%; I^2^ = 71.7%). Three of the five primary cohorts were at high risk of bias. Definitions varied; excluding the largest cohort, which used a broader clinician-suspected joint-symptom definition, yielded a pooled proportion of 2.61% (95% CI, 1.50%-4.79%). Three studies reported 19 PMR/PMR-like events among 1,998 patients (1.08%; 95% CI, 0.32%-3.62%), with marked imprecision and heterogeneity. A 2026 cohort reporting 12 PMR cases among 734 patients was retained as contextual evidence because *de novo* timing could not be verified and possible overlap with an earlier report could not be excluded. Descriptive evidence showed heterogeneous phenotypes and disease courses, with persistence in some cohorts and remission in others. Glucocorticoids and disease-modifying antirheumatic drugs were frequently used, but comparative treatment effects could not be determined.

**Conclusion:**

In denominator-valid studies, approximately 3% of ICI-treated patients were classified as having *de novo* ICI-IA during variable study-specific follow-up. This is a crude study-period proportion, not a fixed-time incidence risk. Standardized definitions and prospective denominator-valid cohorts are needed.

**Systematic review registration:**

https://www.crd.york.ac.uk/PROSPERO/view/CRD420261460334, identifier CRD420261460334.

## 1 Introduction

Immune checkpoint inhibitors have transformed the treatment of many early-stage and advanced cancers by restoring antitumor T-cell activity and producing durable responses in selected patients (1). The same immune activation can disrupt peripheral tolerance and cause immune-related adverse events in almost any organ system (2–4). Although musculoskeletal events are generally less immediately life-threatening than myocarditis, pneumonitis, or neurologic toxicity, inflammatory arthritis and polymyalgia rheumatica (PMR)-like syndromes can impair function, persist after immunotherapy is stopped, and require prolonged immunomodulatory treatment.

Rheumatic immune-related adverse events present a particular management challenge because inflammation must be controlled without unnecessarily compromising cancer therapy. Current rheumatology, oncology, and multidisciplinary guidance recommends phenotype-directed assessment, graded anti-inflammatory treatment, and individualized decisions about continuing or withholding immunotherapy (5–7). The evidence informing these recommendations is heterogeneous. Some studies begin with an identifiable ICI-treated source cohort, whereas others describe patients referred to rheumatology after symptoms have developed. The former can inform occurrence when both the numerator and denominator are valid; the latter provide more detailed accounts of phenotype, persistence, and treatment but cannot estimate population frequency.

Previous reviews have summarized ICI-associated musculoskeletal manifestations and inflammatory arthritis (8, 9). Separate reviews have quantified trial-reported arthralgia and myalgia or described PMR-like phenotypes and their management (10–12). These reviews establish the breadth of rheumatic toxicity but address related yet distinct estimands. Trial reports often capture CTCAE-defined arthralgia or myalgia without adjudicated inflammatory arthritis, whereas referral cohorts and case reports provide detailed clinical descriptions without a denominator representing the ICI-treated population. Frequency estimates are further complicated when objective synovitis, rheumatologist-confirmed inflammatory arthritis, clinician-suspected arthritis, inflammatory arthralgia, and PMR-like presentations are grouped under similar labels, and when follow-up windows are incompatible. Denominator-valid occurrence estimates should therefore be considered separately from clinical-course evidence derived from selected rheumatology populations.

Accordingly, we conducted a systematic review and meta-analysis to estimate the crude occurrence proportion of study-defined *de novo* ICI-IA in denominator-valid ICI-treated source cohorts. Secondary objectives were to examine the occurrence of PMR/PMR-like syndromes and to summarize phenotype, persistence, treatment, and management of ICI therapy as complementary clinical-course evidence. Quantitative pooling was limited to clinically and statistically compatible estimands; evidence that could not be pooled was summarized descriptively. The review was registered and is publicly available in PROSPERO (CRD420261460334).

## 2 Methods

### 2.1 Protocol, registration, and reporting

This systematic review and meta-analysis was conducted and reported in accordance with PRISMA 2020 (13). The review was registered in PROSPERO on July 24, 2026 (CRD420261460334). Evidence that could not be combined without altering its clinical or statistical meaning was synthesized in accordance with the Synthesis Without Meta-analysis (SWiM) reporting framework (14).

### 2.2 Eligibility criteria

We framed the review question using PICOS. The population comprised adults with cancer who had received at least one immune checkpoint inhibitor, including programmed cell death protein 1, programmed death-ligand 1, cytotoxic T-lymphocyte-associated protein 4, or another checkpoint-targeted agent. ICI treatment was the intervention or exposure. No comparator was required for single-group occurrence estimates. The primary outcome was study-defined *de novo* ICI-IA. Secondary outcomes were study-defined *de novo* ICI-PMR/PMR-like syndrome and, when sufficiently comparable, use of systemic glucocorticoids or conventional synthetic disease-modifying antirheumatic drugs (csDMARDs). Clinical phenotype, persistence, remission or relapse, and management of ICI therapy were summarized descriptively as complementary clinical-course outcomes. Cancer survival outcomes listed in the PROSPERO record were retained only as contextual evidence and did not determine study eligibility or support quantitative synthesis. Eligible designs included denominator-valid cohort studies for occurrence and defined referral cohorts or consecutive case series for clinical-course outcomes.

To contribute to an occurrence estimate, a report had to describe an identifiable ICI-treated source cohort with a corresponding numerator and denominator. For clinical course and management, defined referral cohorts, consecutive case series, and formally published conference abstracts were eligible when target-phenotype data could be separated. Single case reports, anecdotal nonconsecutive series, reviews, editorials, animal studies, and reports without a separable target outcome were excluded from the synthesis. Flares of pre-existing inflammatory arthritis or PMR were excluded from *de novo* occurrence estimates when they could be distinguished from new-onset disease in the source report. For occurrence cohorts, baseline rheumatic disease, timing of onset, and the separability of pre-existing flares from *de novo* events were recorded when reported. Noninflammatory arthralgia, myalgia, crystal arthritis, infection, metastatic disease, and other alternative explanations were excluded unless the source investigators explicitly adjudicated ICI-IA or PMR-like disease. During the revision, the 135 previously unclassified reports were rechecked at the report level. Reports that did not provide a source-verifiable *de novo* numerator/denominator pair or a separable target phenotype were retained only for reconciliation and were not included in quantitative synthesis.

We classified case definitions according to the strength of ascertainment. Tier A required confirmation by a rheumatologist or other specialist together with objective inflammatory findings. Tier B included clinician-attributed ICI-related inflammatory arthritis or PMR-like disease when objective documentation was incomplete. Tier C comprised code-based or label-only definitions and was restricted to sensitivity analysis or structured narrative synthesis. PMR and PMR-like features were interpreted with reference to the 2012 EULAR/ACR classification framework, while recognizing that these criteria were developed for primary PMR rather than ICI-associated syndromes (15). No restrictions were imposed by language or publication year, and formally published conference abstracts were eligible.

### 2.3 Information sources and search strategy

Following protocol registration, PubMed, Ovid MEDLINE, Embase, Web of Science Core Collection, and Cochrane CENTRAL were systematically searched from database inception to July 25, 2026. The search strategy combined controlled vocabulary and free-text terms related to immune checkpoint inhibitors, inflammatory arthritis, synovitis, polymyalgia rheumatica, PMR-like syndromes, and rheumatic immune-related adverse events. Reference lists of included studies and relevant reviews were screened to identify additional eligible reports. The available search concepts and database coverage are summarized in **Supplementary Methods S1**; complete database-specific line-by-line search histories were not retained.

### 2.4 Study selection

Two reviewers independently screened titles and abstracts and then assessed potentially eligible full texts. Disagreements were resolved through discussion and, when necessary, by a third reviewer. Reports arising from the same underlying cohort were linked before synthesis. Eligibility was determined at the report level, whereas quantitative analyses were conducted at the level of the independent study or cohort. The 135-report historical reconciliation subset was not treated as an additional PRISMA screening stage; it was included within the final report-level eligibility assessment. The report-level reconciliation record was retained by the authors and can be supplied upon reasonable request.

### 2.5 Data extraction and management of overlapping reports

Two reviewers independently extracted the data and cross-checked the final extraction records. A third reviewer resolved disagreements. Extracted items included study setting and design, cancer population, ICI regimen, recruitment period, source-cohort denominator, target-event numerator, case definition, ascertainment route, follow-up frame, phenotype, disease course, treatment exposure, ICI management, oncologic outcomes when reported, baseline rheumatic disease, separability of pre-existing flares from *de novo* events, PMR assessment status, age distribution, anti-CTLA-4-containing combination therapy, and source location. Reports from a shared cohort were assigned a common study-family identifier. For each estimand, we selected the report with the clearest case definition, most complete denominator, and most informative follow-up; companion reports were used only for nonoverlapping outcomes or time points.

### 2.6 Risk-of-bias assessment

Two reviewers independently assessed risk of bias using design-matched Joanna Briggs Institute checklists (16) for cohort studies, case series, prevalence studies, case-control studies, and analytical cross-sectional studies. A third reviewer resolved disagreements. Judgments considered source-population representativeness, exposure and outcome ascertainment, exclusion of pre-existing disease for *de novo* outcomes, denominator validity, follow-up completeness, confounding, and appropriateness of the statistical analysis. Risk-of-bias findings were summarized for reports contributing to the quantitative or SWiM syntheses. Outcome-level certainty judgments are presented in **Supplementary Table S3**.

### 2.7 Quantitative synthesis

The principal estimand was the crude proportion of ICI-treated patients classified as having study-defined *de novo* ICI-IA during the follow-up available in each study. Because follow-up duration and ascertainment windows differed, we treated this estimand as a crude study-period occurrence proportion rather than an annual incidence rate, fixed-time cumulative risk, or person-time incidence rate. Cumulative incidence estimates and person-time rates were not combined with crude proportions.

Single-group proportions were synthesized with a random-intercept binomial generalized linear mixed model using a logit link (17). Study-level confidence intervals were calculated by the Clopper-Pearson method (18), and no continuity correction was applied in the primary model. Random-effects pooled estimates were reported with 95% confidence intervals (CIs), 95% prediction intervals when estimable, I^2^, and τ^2^. We repeated the primary ICI-IA analysis after excluding the conference abstract, excluding the Tran cohort, substituting the imaging-supported numerator for Buder-Bakhaya, and adding one lower-detail source cohort. PMR/PMR-like occurrence, csDMARD use, and systemic glucocorticoid use were treated as exploratory quantitative syntheses because few studies contributed and the underlying populations were selected. Anti-CTLA-4-containing combination therapy was assessed descriptively because regimen proportions were not consistently available across the occurrence cohorts. A separate contextual synthesis pooled CTCAE-defined all-grade arthralgia reported in melanoma ICI trial arms; these data were not combined with ICI-IA because arthralgia and inflammatory arthritis represented different outcomes. An age-restricted PMR analysis (≥50 years) was planned if age-specific event counts and corresponding ICI-treated denominators were available.

No subgroup analyses were planned or performed because study-level covariates and common outcome definitions were insufficient. Funnel-plot asymmetry tests, Egger regression, and trim-and-fill were not applied because each quantitative synthesis included fewer than 10 studies and involved sparse single-arm proportions. All statistical analyses were performed using R version 4.6.1 (R Foundation for Statistical Computing, Vienna, Austria) following established R-based meta-analytic procedures (19, 20).

### 2.8 Structured synthesis without meta-analysis

Clinical-course evidence that could not be pooled was summarized descriptively as a complement to the meta-analysis. The summary addressed measurement, phenotype, disease course, treatment use, and management of ICI therapy. We did not use vote counting or draw comparative treatment-effect inferences. Cancer survival outcomes listed in the PROSPERO record were considered separately as contextual evidence because no common estimand could be defined.

## 3 Results

### 3.1 Study selection and analysis contribution

The searches identified 15,665 records. After title/abstract screening and retrieval reconciliation, 840 reports were assessed for eligibility. In total, 775 reports were excluded, and 65 reports representing 63 independent studies were included in the final evidence set (**Figure 1**). The 135-report historical reconciliation subset was part of these report-level exclusions and was not counted separately.

**Figure 1:**
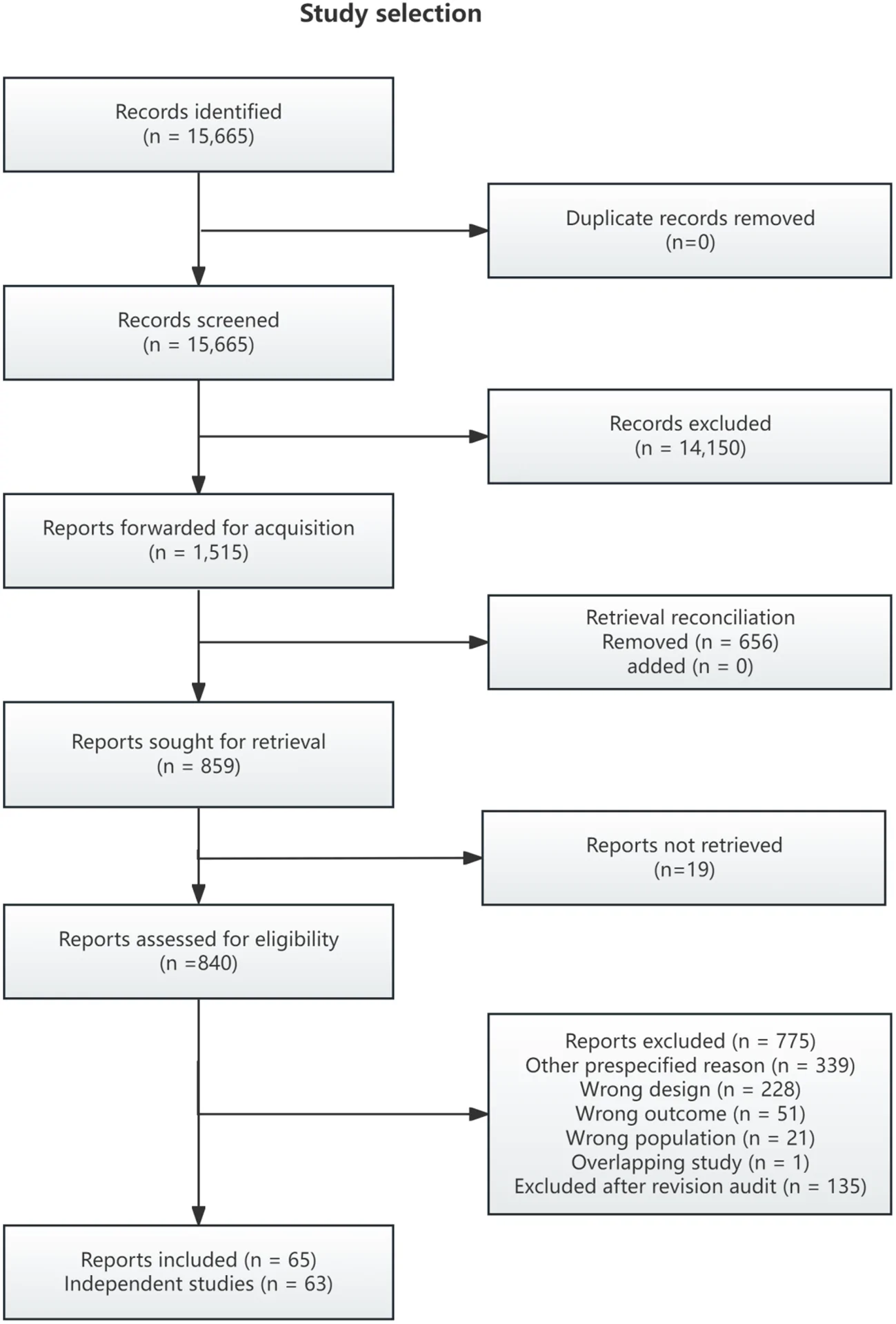
Study selection. Final PRISMA 2020 flow diagram based on the final screening counts: 15,665 records screened; 14,150 title/abstract records excluded; 840 full-text reports assessed; 775 full-text reports excluded after report-level adjudication; and 65 reports representing 63 studies retained for synthesis. The historical 135-report reconciliation subset was included within the 775 full-text exclusions and was not an additional flow stage.

Of the 65 included reports, eight contributed to at least one overall or sensitivity meta-analysis, 61 contributed to at least one SWiM theme, and four contributed to both. Study characteristics and the role of each report in the synthesis are detailed in **Supplementary Table S1**. Additional included reports are detailed in Supplementary Table S1 (21–50).

### 3.2 Characteristics of the primary occurrence evidence

Five independent real-world ICI-treated source cohorts contributed to the primary ICI-IA occurrence synthesis (**Table 1**). Conducted in Germany, France, the United States, and Spain, they included prospective, retrospective, and electronic health record designs. Case ascertainment ranged from specialist-supported inflammatory findings to broader clinician-suspected ICI-IA, and follow-up was not harmonized across studies. We found no evidence of participant overlap among the five cohorts.

**Table 1:** Characteristics of the five cohorts in the primary ICI-IA occurrence meta-analysis.

Study	Setting/design	ICI cohort, n	ICI-IA, n	Case definition	Source
Buder-Bakhaya 2018 (51)	Germany; retrospective PD-1 cohort	195	10	Clinically classified arthritis; 7/10 imaging-supported; Tier B	Full article
Kostine 2018 (52)	France; prospective single-center cohort	524	9*	Inflammatory arthritis after separating PMR; mixed objective confirmation; Tier B	Full article
Richter 2019 (53)	United States; retrospective single-center cohort	1,293	25†	Source-cohort cases with synovitis features; Tier B/mixed	Full article
Lopez Gonzalez 2023 (54)	Spain; prospective multidisciplinary cohort	181	7	Rheumatology-evaluated cases; limited case-level detail; Tier B/unclear	Conference abstract
Tran 2024 (55)	United States; retrospective EHR cohort	2,451	89	Clinician-suspected ICI-related joint pain, arthralgia, or arthritis; Tier B	Full article

EHR, electronic health record; ICI, immune checkpoint inhibitor; ICI-IA, immune checkpoint inhibitor-associated inflammatory arthritis; PMR, polymyalgia rheumatica. Follow-up and risk windows varied across cohorts; therefore, the pooled estimand is a study-period crude occurrence proportion. Anti-CTLA-4-containing combination therapy was derivable only for Buder-Bakhaya (36/195, 18.5%); corresponding proportions were not reported or not derivable for Kostine, Richter, López González, and Tran. *Kostine reported 20 inflammatory rheumatic presentations, comprising 7 rheumatoid arthritis-like, 11 PMR-like, and 2 psoriatic arthritis-like cases; the 11 PMR-like cases were analyzed separately, leaving 9 non-PMR inflammatory arthritis cases for the ICI-IA occurrence analysis. †Richter reported 34 inflammatory arthritis cases in the overall 61-patient rheumatology series; 25 IA cases were mapped to the denominator-valid 1,293-patient source cohort used for occurrence estimation.

### 3.3 Primary meta-analysis of *de novo* ICI-IA

The five cohorts reported 140 study-defined *de novo* ICI-IA events among 4,644 ICI-treated patients (**Table 1**). The pooled crude occurrence proportion was 2.87% (95% CI 2.02%-4.07%), with a 95% prediction interval of 1.06%-7.53%. Between-study heterogeneity was substantial (I^2^ = 71.7%; τ^2^ = 0.099 on the fitted model scale) (**Figure 2**). Study-level data were 10/195 for Buder-Bakhaya, 9/524 for Kostine, 25/1,293 for Richter, 7/181 for López González, and 89/2,451 for Tran. In Kostine et al., 20 inflammatory rheumatic presentations comprised 7 rheumatoid arthritis-like, 11 PMR-like, and 2 psoriatic arthritis-like cases; the 11 PMR-like cases were analyzed separately, leaving 9 non-PMR inflammatory arthritis cases for the ICI-IA analysis. In Richter et al., 34 inflammatory arthritis cases were described in the overall 61-patient rheumatology series, whereas 25 were mapped to the denominator-defined 1,293-patient source cohort used for occurrence estimation.

**Figure 2:**
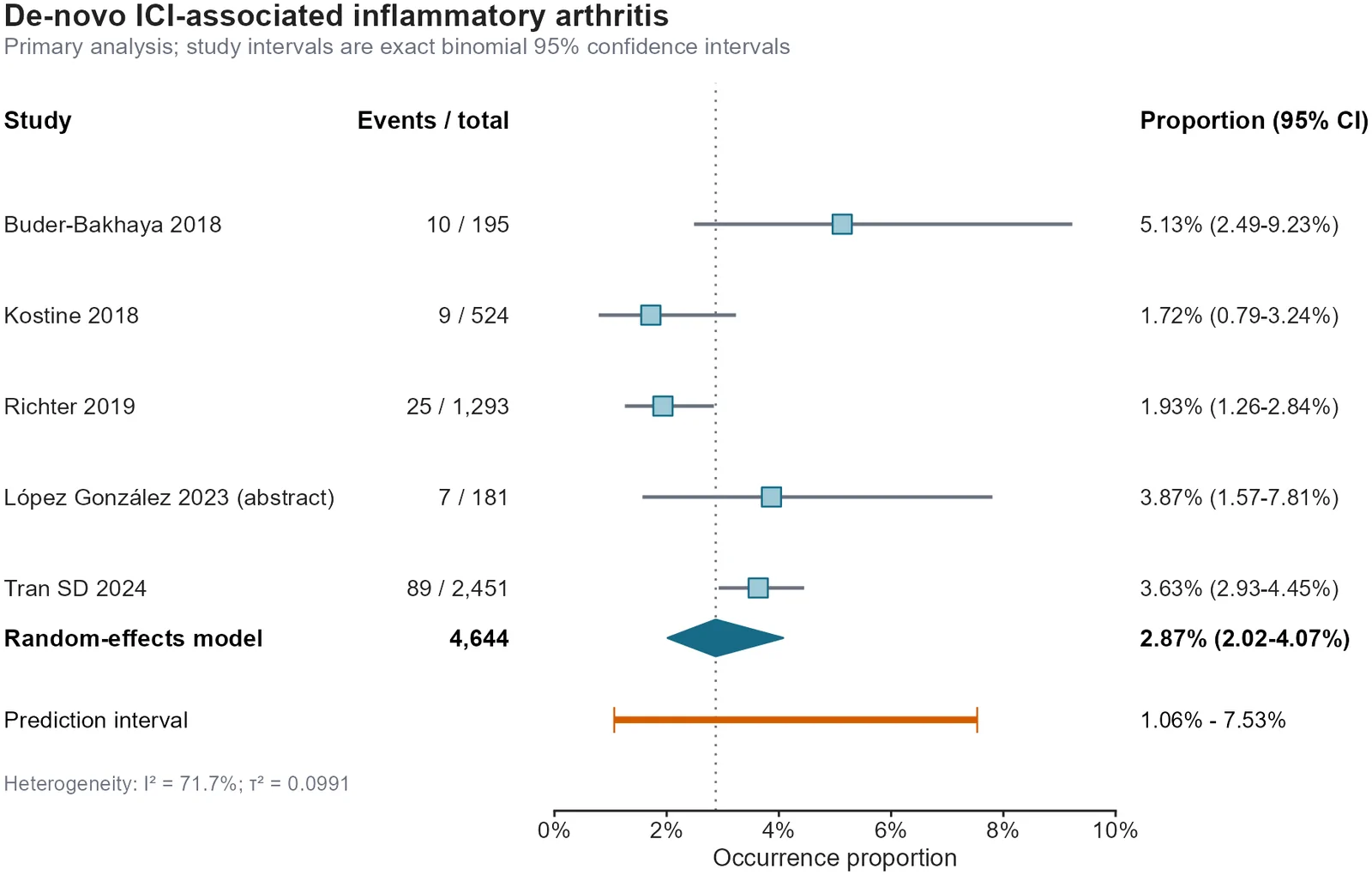
Meta-analysis of *de novo* ICI-associated inflammatory arthritis. Study-specific proportions are shown with exact 95% confidence intervals. The diamond represents the random-intercept binomial GLMM estimate, and the orange line represents the 95% prediction interval.

Thus, approximately 3% of ICI-treated patients were classified as having *de novo* ICI-IA during variable, study-specific follow-up. This estimate should not be interpreted as an annual incidence rate, a common fixed-time cumulative risk, or an individual patient’s predicted risk.

### 3.4 Sensitivity analyses for *de novo* ICI-IA

The central estimate remained broadly similar across the sensitivity analyses (**Figure 3**). Excluding the Tran cohort yielded 51 events among 2,193 patients and a pooled proportion of 2.61% (95% CI 1.50%-4.79%; 95% prediction interval 1.09%-6.13%; I^2^ = 78.0%). Excluding the conference abstract (54) yielded 133 events among 4,463 patients and a pooled proportion of 2.76% (95% CI 1.85%-4.09%; 95% prediction interval 0.79%-9.15%; I^2^ = 78.4%). Adding one lower-detail source cohort (56) yielded 143 events among 4,774 patients and a pooled proportion of 2.82% (95% CI 2.04%-3.90%; 95% prediction interval 1.19%-6.53%; I^2^ = 65.4%). Restricting the Buder-Bakhaya numerator to seven imaging-supported cases (51) yielded 137 events among 4,644 patients and a pooled proportion of 2.72% (95% CI 1.97%-3.73%; 95% prediction interval 1.16%-6.23%; I^2^ = 66.0%).

**Figure 3:**
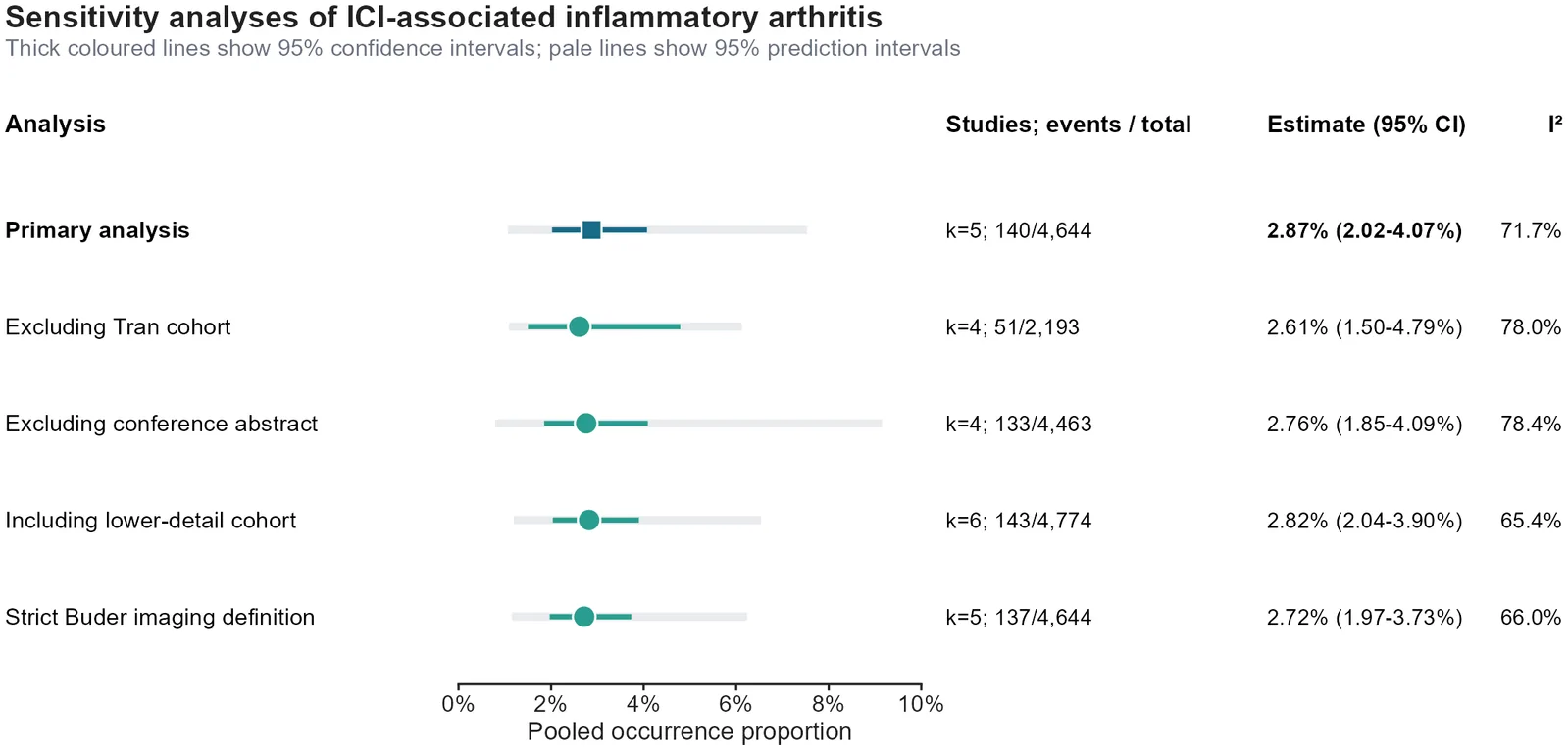
Sensitivity analyses of ICI-associated inflammatory arthritis. Points and colored lines show pooled proportions and 95% confidence intervals; pale lines show 95% prediction intervals.

Pooled point estimates ranged from 2.61% to 2.82% across these analyses. Excluding Tran modestly lowered the estimate, consistent with the influence of its broader case definition, but did not eliminate substantial heterogeneity. The other sensitivity analyses likewise did not materially change the central estimate.

### 3.5 Exploratory PMR/PMR-like occurrence

Three studies reported 19 study-defined *de novo* PMR/PMR-like events among 1,998 ICI-treated patients (52–54). The exploratory pooled crude occurrence proportion was 1.08% (95% CI 0.32%-3.62%), with a 95% prediction interval of 0.01%-63.42% and substantial heterogeneity (I^2^ = 85.4%) (**Figure 4A**). Confirmed zero events were distinguished from outcomes that were not assessed or not separately reported; no additional confirmed denominator-valid zero-event cohort was available for the primary PMR pool. Age-specific PMR numerators and denominators were not consistently reported, so a valid analysis restricted to patients aged ≥50 years could not be performed. The very wide prediction interval underscores the uncertainty across settings. The 2026 Llobell-Uriel cohort was retained as contextual clinical-course evidence rather than added to the primary pooled estimate because case-level *de novo* timing and possible overlap with the 2025 POS0859 report could not be verified from the available report-level record (57, 58).

**Figure 4:**
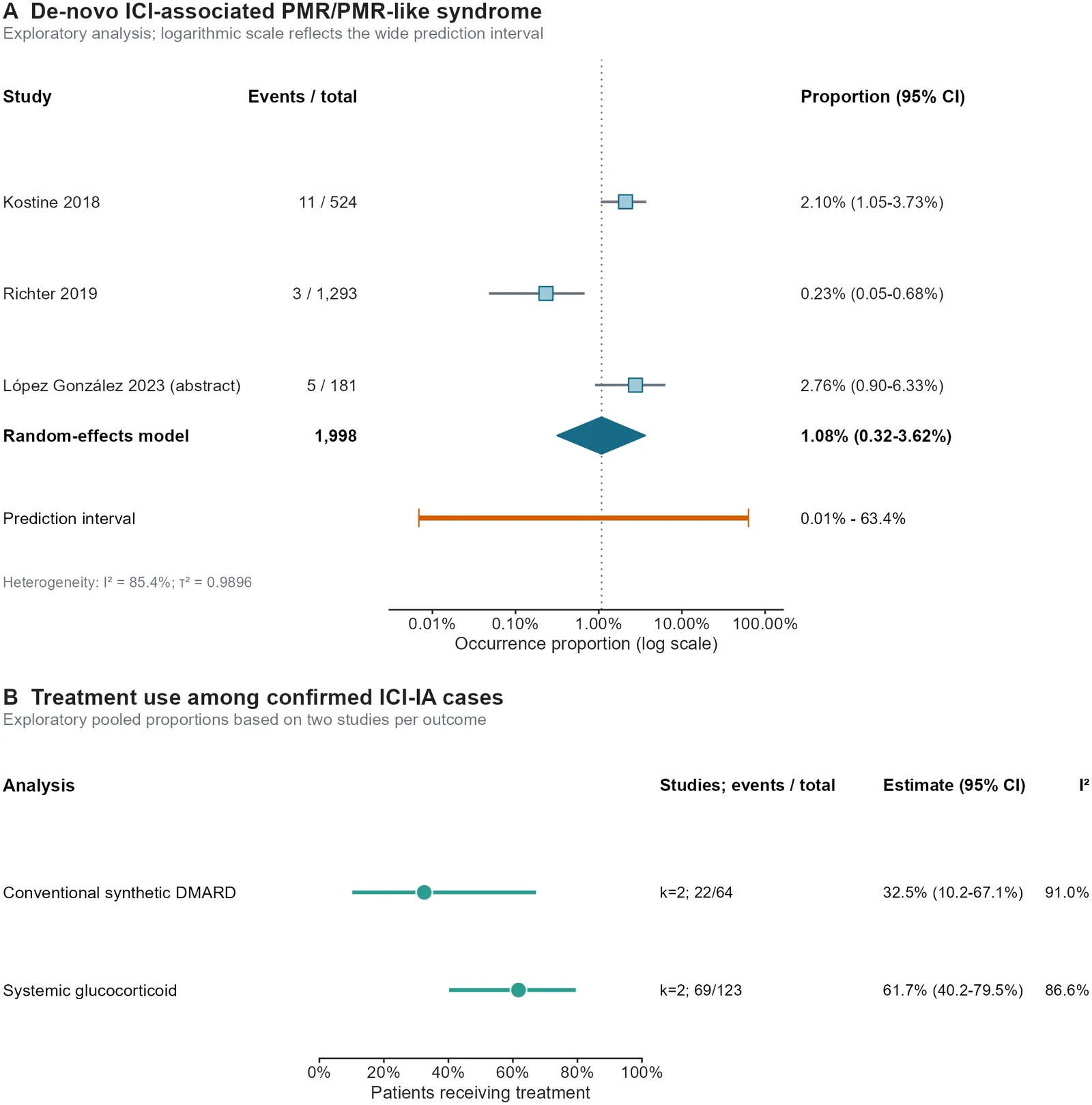
Exploratory meta-analyses. **(A)** *De novo* ICI-associated PMR/PMR-like syndrome. **(B)** Use of conventional synthetic DMARDs and systemic glucocorticoids among confirmed ICI-IA cases. These estimates are exploratory because few studies contributed and heterogeneity was substantial.

After exclusion of the conference abstract (54), two studies with 14 events among 1,817 patients yielded a pooled proportion of 0.71% (95% CI 0.15%-3.36%; I^2^ = 91.3%). Adding one lower-detail source cohort (59) produced 21 events among 2,494 patients and a pooled proportion of 0.85% (95% CI 0.29%-2.44%; 95% prediction interval 0.03%-22.61%; I^2^ = 82.5%) (**Supplementary Figures S1, S2**). These exploratory estimates do not provide a precise measure of population frequency.

### 3.6 Contextual melanoma-trial arthralgia analysis

All-grade CTCAE arthralgia counts were extracted from **Table 2** of Roberts et al. (10). Four phase III melanoma trials contributed: IMspire150 and COMBI-i combined an ICI with BRAF/MEK inhibitors (61, 62). KEYNOTE-054 and KEYNOTE-716 evaluated adjuvant pembrolizumab (63, 64). The ICI-containing arms reported 103/231, 62/267, 64/509, and 115/483 patients with arthralgia, respectively (1,490 patients in total). Using the same random-intercept binomial GLMM, the pooled arthralgia occurrence proportion was 24.57% (95% CI 11.85%-43.47%; 95% prediction interval 7.05%-58.33%; I^2^ = 64.2%). These data were not pooled with the real-world ICI-IA cohorts because the outcome definitions, treatment regimens, populations, and observation periods differed. Arthritis was not separately reported in the adverse-event data summarized by Roberts et al.; this does not establish that no arthritis occurred (**Supplementary Table S6**). The pooled proportion describes arthralgia in ICI-containing treatment arms and does not isolate an ICI-attributable effect.

**Table 2:** Quantitative synthesis results.

Analysis	k	Events/N	Pooled proportion	95% CI	95% prediction interval	I^2^
Primary ICI-IA (51–55)	5	140/4,644	2.87%	2.02%-4.07%	1.06%-7.53%	71.7%
ICI-IA: exclude Tran cohort (51–54)	4	51/2,193	2.61%	1.50%-4.79%	1.09%-6.13%	78.0%
ICI-IA: exclude conference abstract (51–53, 55)	4	133/4,463	2.76%	1.85%-4.09%	0.79%-9.15%	78.4%
ICI-IA: add lower-detail cohort (51–56)	6	143/4,774	2.82%	2.04%-3.90%	1.19%-6.53%	65.4%
ICI-IA: strict imaging numerator (51–55)	5	137/4,644	2.72%	1.97%-3.73%	1.16%-6.23%	66.0%
PMR/PMR-like overall (52–54)	3	19/1,998	1.08%	0.32%-3.62%	0.01%-63.42%	85.4%
PMR/PMR-like: exclude conference abstract (52, 53)	2	14/1,817	0.71%	0.15%-3.36%	Not estimable	91.3%
PMR/PMR-like: add lower-detail cohort (52–54, 59)	4	21/2,494	0.85%	0.29%-2.44%	0.03%-22.61%	82.5%
Melanoma-trial arthralgia (ICI arms) (10)	4	344/1,490	24.57%	11.85%-43.47%	7.05%-58.33%	64.2%
csDMARD use (53, 60)	2	22/64	32.52%	10.20%-67.15%	Not estimable	91.0%
Systemic glucocorticoid use (53, 55)	2	69/123	61.74%	40.20%-79.48%	Not estimable	86.6%

All estimates used random-intercept binomial GLMMs with a logit link. Treatment-use and PMR/PMR-like syntheses were exploratory. The melanoma-trial arthralgia synthesis was contextual and was not pooled with ICI-IA. CI, confidence interval; csDMARD, conventional synthetic disease-modifying antirheumatic drug; ICI-IA, immune checkpoint inhibitor-associated inflammatory arthritis; PMR, polymyalgia rheumatica.

### 3.7 Exploratory treatment-use syntheses

Two phenotype-defined cohorts reported csDMARD use in 22 of 64 patients (53, 60). The exploratory pooled proportion was 32.52% (95% CI 10.20%-67.15%; I^2^ = 91.0%) (**Figure 4B**; **Supplementary Figure S3**). Two cohorts reported systemic glucocorticoid use in 69 of 123 patients (53, 55), with a pooled proportion of 61.74% (95% CI 40.20%-79.48%; I^2^ = 86.6%) (**Figure 4B**; **Supplementary Figure S4**). These estimates describe treatment use in selected clinical cohorts and should not be interpreted as comparative treatment effects or preferred management strategies.

### 3.8 Structured clinical synthesis

Sixty-one reports representing 59 independent studies supplied non-poolable evidence for the complementary synthesis (**Supplementary Table S1**). Reported presentations included peripheral arthritis, PMR-like, and overlap phenotypes, with both persistence and remission described over different follow-up periods (65, 66). Systemic glucocorticoids were used frequently, whereas DMARD escalation was described mainly in selected patients with persistent or treatment-refractory disease. Decisions to continue, interrupt, discontinue, or rechallenge ICI therapy varied with the clinical context (67, 68). Differences in definitions, time origins, follow-up windows, and patient selection precluded additional pooled frequency estimates or comparative treatment-effect conclusions (**Supplementary Table S4**). Four studies also reported the cancer survival outcomes listed in the registered record (60, 69). Incompatible cancer types, endpoints, timing, and comparator structures prevented any inference of oncologic benefit or harm (70, 71).

### 3.9 Risk of bias

Of the 65 reports contributing to the quantitative or SWiM syntheses, 33 had moderate risk of bias and 32 had high risk. The five primary occurrence cohorts included two at moderate risk and three at high risk (**Table 3**). Common concerns were incomplete reporting of follow-up, broad or incompletely documented case ascertainment, limited control of confounding, and selection of referral or other clinical populations. These limitations are consistent with the substantial heterogeneity and support interpreting the pooled result as an approximate study-period occurrence proportion.

**Table 3:** Risk-of-bias judgments for the five primary ICI-IA occurrence cohorts.

Study	Tool	Overall judgment	Main concerns
Buder-Bakhaya 2018 (51)	JBI cohort	High	Incomplete reporting of ascertainment safeguards and follow-up completeness; only 7/10 cases were imaging-supported.
Kostine 2018 (52)	JBI cohort	Moderate	Mixed objective confirmation and incomplete reporting of baseline exclusion and follow-up completeness.
Richter 2019 (53)	JBI cohort	High	Limited reporting of follow-up completeness, confounding, and statistical safeguards for the source-cohort estimate.
Lopez Gonzalez 2023 (54)	JBI cohort	High	Conference-abstract source with limited case-level ascertainment and follow-up detail.
Tran 2024 (55)	JBI cohort	Moderate	Broad clinician-suspected case definition and incomplete reporting of follow-up completeness.

ICI-IA, immune checkpoint inhibitor-associated inflammatory arthritis; JBI, Joanna Briggs Institute. Judgments apply to the occurrence estimates used in the primary synthesis.

No subgroup analysis was performed, and formal testing for small-study effects was not undertaken because the quantitative syntheses were too sparse for reliable inference.

## 4 Discussion

Among verified studies with valid denominators, approximately 3% of ICI-treated patients were classified as having study-defined *de novo* inflammatory arthritis during the follow-up available in each study. The point estimate changed little after exclusion of the conference abstract, addition of a lower-detail cohort, or use of a stricter imaging-supported numerator; exclusion of Tran yielded 2.61% and modestly lowered the estimate. These findings suggest that no single analytic decision fully drove the central estimate, although broad definitions and heterogeneity remain important. This stability does not make the result a fixed-time cumulative incidence or a person-time rate. The wide prediction interval and substantial between-study heterogeneity indicate that occurrence may vary considerably across clinical settings and ascertainment pathways.

Occurrence and clinical course were informed by different evidence structures. Denominator-valid source cohorts could estimate a crude study-period occurrence proportion, whereas referral cohorts and case series provided most of the evidence on phenotype, persistence, treatment escalation, and ICI interruption or rechallenge. PMR/PMR-like occurrence and treatment-use estimates were based on few studies and remained exploratory. The structured synthesis therefore served as a complement to the meta-analysis, not as an additional frequency calculation.

Earlier reviews have described the breadth and management of ICI-associated musculoskeletal toxicity (8, 9). The melanoma trial meta-analysis by Roberts et al. quantified arthralgia and myalgia but did not report arthritis as a trial outcome (10). In the present contextual synthesis, CTCAE-defined all-grade arthralgia in melanoma ICI arms was substantially more frequent than study-defined ICI-IA, but the two outcomes represent different clinical constructs and were not combined. Case-based reviews of PMR-like syndromes noted inconsistent use of classification criteria and limited evidence on ICI continuation or rechallenge (11, 12). By restricting the primary pool to identifiable ICI-treated source cohorts and separating these data from referral-based clinical-course evidence, the present analysis estimates a different quantity. Even among the five primary cohorts, recruitment setting, case ascertainment, and the clinical boundaries between inflammatory arthritis, arthralgia, and PMR-like disease varied (**Table 1**). The pooled result is therefore best interpreted as the proportion classified under study-specific real-world definitions, not the frequency of a uniformly adjudicated disease entity.

Clinical and imaging evidence indicates biologic and phenotypic diversity that is unlikely to reflect measurement error alone. Peripheral polyarthritis, oligoarthritis, large-joint disease, enthesitis, tenosynovitis, axial symptoms, activated osteoarthritis, and PMR-like presentations have all been described. Whole-body MRI and ultrasound studies have documented synovial, tendon, entheseal, and periarticular inflammation (72–74). Latent-class and translational studies further suggest that ICI-associated inflammatory arthritis may comprise more than one clinical-immunologic pattern (75–77). Early case series likewise showed that checkpoint blockade can produce inflammatory joint syndromes that do not fit neatly within conventional rheumatoid arthritis or spondyloarthritis categories (78, 79). Future cohorts should report phenotype and objective inflammatory features explicitly rather than relying on a single broad arthritis label.

The largest primary cohort contributed 89 of the 140 events and used a broad, clinician-suspected definition. Such definitions may capture early or incompletely evaluated inflammation, but they may also include noninflammatory arthralgia or PMR-like symptoms. Excluding Tran modestly reduced the pooled estimate to 2.61% but did not resolve heterogeneity. The strict Buder-Bakhaya sensitivity analysis examined another definitional boundary and produced a similar pooled estimate; however, no sensitivity analysis could retrospectively harmonize all cases in the largest cohort. A common core definition, accompanied by parallel reporting of broader clinically suspected cases, would improve comparability without excluding patients encountered in routine practice.

The clinical course was not consistently self-limited. Persistent arthritis after ICI discontinuation has been reported in several cohorts (80, 81). Longer follow-up of adjuvant melanoma cohorts also documented chronic immune-related adverse events (82, 83). In contrast, resolution during continued treatment or after withdrawal has also been described (84). These observations are not necessarily contradictory because the studies used different starting points, follow-up periods, treatment exposures, and definitions of remission. Improvement at a single visit and sustained drug-free remission are clinically distinct outcomes. Future studies should report disease activity at prespecified time points, treatment status at each assessment, and whether remission is clinical, imaging-based, or treatment-free.

Glucocorticoids were the most frequently reported systemic treatment, and conventional synthetic disease-modifying antirheumatic drugs were used when symptoms persisted or steroid tapering was difficult (66, 85). Hydroxychloroquine, methotrexate, and other csDMARDs have been described as steroid-sparing approaches (86–88). Tocilizumab and other treatment-escalation strategies have also been reported in selected patients (89–91). Treatment selection was closely related to disease severity and prior response. The pooled treatment-use proportions summarize selected cohorts and do not permit efficacy comparisons. Confounding by indication is unavoidable when patients with more severe or persistent disease preferentially receive stronger immunosuppression. Comparative prospective registries with standardized measures of disease activity and cancer outcomes are needed to move beyond uncontrolled response proportions.

Decisions to continue, temporarily interrupt, permanently discontinue, or rechallenge ICI therapy were made in different clinical contexts. Rechallenge studies suggest that treatment can be resumed in some patients, but those selected for rechallenge had survived, recovered sufficiently, and been judged suitable by their clinical teams (67). This selection precludes a universal rule. Current guideline-based practice remains appropriate and requires arthritis severity, cancer status, alternative oncologic options, and the immunosuppression needed for symptom control to be considered together (5–7).

The pooled PMR/PMR-like proportion was lower than the ICI-IA estimate, but both the confidence and prediction intervals were very wide. Age thresholds, classification features, imaging, and terminology varied across reports, and the distinction between PMR-like disease and inflammatory arthritis was not uniform (52, 57, 92). The 2012 EULAR/ACR classification framework offers useful clinical context but was developed for primary PMR, not immunotherapy-associated syndromes (15). Reports of ICI-associated PMR-like disease should therefore provide the individual classification features, age, inflammatory markers, imaging findings, and presence of peripheral synovitis rather than relying on the diagnostic label alone. The Llobell-Uriel cohort was therefore described separately rather than used to alter the exploratory pooled estimate; this conservative decision was based on report-level verification and possible overlap, not solely on the presence of other autoimmune conditions (58).

For clinicians, the pooled estimate provides an approximate benchmark: inflammatory arthritis is uncommon but not rare among ICI-treated patients, and some may require rheumatology assessment and treatment beyond a short glucocorticoid course. New joint pain should not automatically be labeled inflammatory arthritis. Examination for synovitis, assessment of inflammatory and mechanical alternatives, and targeted imaging can improve classification. Management should remain coordinated between oncology and rheumatology because symptom control, cancer status, ICI continuation, and cumulative immunosuppression are interdependent.

For research, increasing the pooled sample alone is not enough. Denominator-valid cohorts should define the at-risk population, time origin, observation window, incident status, case ascertainment pathway, and handling of death or loss to follow-up. Reports should distinguish confirmed inflammatory arthritis from broader suspected cases and present PMR-like disease separately. Clinical cohorts should use common measures of disease activity, function, treatment response, and remission. These changes would allow future reviews to estimate fixed-time risks, person-time rates, persistence, and comparative treatment outcomes without combining incompatible quantities.

This review linked reports from the same underlying cohort before synthesis and verified the numerator, denominator, case definition, and overlap status of the five primary occurrence cohorts. It also separated outcome-level analytic contributions from the broader evidence base. Quantitative pooling was restricted to compatible single-group proportions, while a structured synthesis retained clinically important evidence that could not be pooled. Sensitivity analyses examined the main source-detail and case-definition boundaries without selectively removing heterogeneous findings.

Several limitations constrain interpretation. First, only five independent cohorts contributed to the primary analysis, and three had high overall risk of bias. Underascertainment is plausible when rheumatic symptoms were not assessed systematically, whereas referral intensity and broad clinical definitions may increase detection; the net direction and magnitude of these biases cannot be quantified. Follow-up periods and observation windows also differed, so the pooled estimate represents a crude study-period occurrence proportion rather than an annual incidence rate or an individual patient’s risk at a specific time point.

Second, the largest cohort contributed 89 of 140 primary events and used a definition that could include arthralgia or PMR-like presentations. Excluding this cohort modestly reduced the pooled estimate but did not resolve heterogeneity, and the analysis remained based on study-defined constructs. Third, the PMR/PMR-like and treatment-use syntheses included only two or three studies, with wide intervals and substantial heterogeneity. Age-specific PMR analyses were not possible because corresponding age-stratified numerators and denominators were unavailable. Anti-CTLA-4 combination proportions were incompletely reported, so regimen effects could be assessed only descriptively. The separate melanoma-trial arthralgia estimate was substantially higher than the ICI-IA estimate but represents a symptom-level CTCAE outcome rather than inflammatory arthritis. These analyses identify signals in the evidence rather than precise population parameters or comparative treatment effects.

Fourth, complete database-specific line-by-line search strings were not retained in the review files, which may limit reproducibility. Cancer survival was listed in the registered record, but the available observational reports used incompatible cancer types, endpoints, time origins, and comparator structures and could not support a pooled oncologic effect estimate (60, 69, 71). Finally, the structured synthesis included multiple outcomes and time points from some study families, and formal funnel-plot asymmetry testing was uninformative because every quantitative synthesis included fewer than 10 studies. Selective publication and selective outcome reporting remain possible. The report-level decision for the separate 2026 Llobell-Uriel cohort is documented in **Supplementary Table S5** (58).

## 5 Conclusion

Among verified cohorts with valid denominators, approximately 3% of ICI-treated patients were classified as having study-defined *de novo* inflammatory arthritis during variable real-world follow-up. This is an approximate crude study-period occurrence proportion, not a fixed-time incidence risk. The occurrence of PMR-like syndromes, comparative treatment effectiveness, and optimal ICI management remain uncertain. More precise time-specific and treatment-relevant estimates will require standardized definitions, explicit observation windows, and prospective denominator-valid cohorts.

## Data Availability

The original contributions presented in the study are included in the article/**Supplementary Material**. Further inquiries can be directed to the corresponding author.
