# Supplementary material for: Occurrence and clinical course of immune checkpoint inhibitor-associated inflammatory arthritis and polymyalgia rheumatica-like syndromes: a systematic review and meta-analysis
Source: Front Immunol. 2026 Sep 30;17:1959233. doi: 10.3389/fimmu.2026.1959233 (PMC13662647; doi:10.3389/fimmu.2026.1959233)
Supplement: Supplementary file 1 [file SupplementaryFile1.docx]

**Supplementary Material**

# Supplementary Methods S1. Search concepts and database coverage

PubMed, Ovid MEDLINE, Embase, Web of Science Core Collection, and Cochrane CENTRAL were searched from database inception to July 25, 2026. The search combined controlled vocabulary and free-text terms related to immune checkpoint inhibitors, inflammatory arthritis, synovitis, polymyalgia rheumatica, PMR-like syndromes, and rheumatic immune-related adverse events. The table below summarizes the available concepts and database coverage. It is not a verbatim record of executed database-specific searches; complete line-by-line search histories were not retained.

| **Database** | **Platform** | **Coverage** | **Search concepts** | **Limits** |
| --- | --- | --- | --- | --- |
| PubMed | PubMed | Inception to 2026-07-25 | ("immune checkpoint inhibitor*" OR ipilimumab OR nivolumab OR pembrolizumab OR atezolizumab OR durvalumab OR avelumab OR cemiplimab OR "PD-1" OR "PD-L1" OR "CTLA-4") AND ("inflammatory arthritis" OR arthritis OR synovitis OR "polymyalgia rheumatica" OR "PMR-like" OR "rheumatic immune-related adverse event*" OR "musculoskeletal immune-related adverse event*") | No language or year restriction documented |
| MEDLINE | Ovid MEDLINE | Inception to 2026-07-25 | ("immune checkpoint inhibitor*" OR ipilimumab OR nivolumab OR pembrolizumab OR atezolizumab OR durvalumab OR avelumab OR cemiplimab OR "PD-1" OR "PD-L1" OR "CTLA-4") AND ("inflammatory arthritis" OR arthritis OR synovitis OR "polymyalgia rheumatica" OR "PMR-like" OR "rheumatic immune-related adverse event*" OR "musculoskeletal immune-related adverse event*") | No language or year restriction documented |
| Embase | Embase | Inception to 2026-07-25 | ("immune checkpoint inhibitor*" OR ipilimumab OR nivolumab OR pembrolizumab OR atezolizumab OR durvalumab OR avelumab OR cemiplimab OR "PD-1" OR "PD-L1" OR "CTLA-4") AND ("inflammatory arthritis" OR arthritis OR synovitis OR "polymyalgia rheumatica" OR "PMR-like" OR "rheumatic immune-related adverse event*" OR "musculoskeletal immune-related adverse event*") | No language or year restriction documented |
| Web of Science Core Collection | Web of Science | Inception to 2026-07-25 | ("immune checkpoint inhibitor*" OR ipilimumab OR nivolumab OR pembrolizumab OR atezolizumab OR durvalumab OR avelumab OR cemiplimab OR "PD-1" OR "PD-L1" OR "CTLA-4") AND ("inflammatory arthritis" OR arthritis OR synovitis OR "polymyalgia rheumatica" OR "PMR-like" OR "rheumatic immune-related adverse event*" OR "musculoskeletal immune-related adverse event*") | No language or year restriction documented |
| Cochrane CENTRAL | Cochrane Library | Inception to 2026-07-25 | ("immune checkpoint inhibitor*" OR ipilimumab OR nivolumab OR pembrolizumab OR atezolizumab OR durvalumab OR avelumab OR cemiplimab OR "PD-1" OR "PD-L1" OR "CTLA-4") AND ("inflammatory arthritis" OR arthritis OR synovitis OR "polymyalgia rheumatica" OR "PMR-like" OR "rheumatic immune-related adverse event*" OR "musculoskeletal immune-related adverse event*") | No language or year restriction documented |

Reference lists of included studies and relevant reviews were screened for additional reports.

# Supplementary Table S1. Characteristics of the 65 included reports

*Rows are reports; Study family labels identify reports arising from the same independent study. NR, not reported; ICI, immune checkpoint inhibitor; IA, inflammatory arthritis; PMR, polymyalgia rheumatica. Overall risk of bias: 33 moderate and 32 high. Reference numbers correspond to the main manuscript reference list. All 65 report rows are retained.*

| **Ref.** | **Family** | **Study** | **Report title** | **Year** | **Country** | **Design** | **Report role** | **Original n** | **ICI n** | **IA n** | **PMR n** | **RoB** |
| --- | --- | --- | --- | --- | --- | --- | --- | --- | --- | --- | --- | --- |
| [21] | S01 | Buder-Bakhaya 2018 | Characterization of arthralgia induced by PD-1 antibody treatment in patients with metastasized cutaneous malignancies. | 2018 | Germany | Retrospective cohort | Primary report | 195 | 195 | 10 | NR | High |
| [22] | S02 | Kostine 2018 | Rheumatic disorders associated with immune checkpoint inhibitors in patients with cancer-clinical aspects and relationship with tumour response: a single-centre prospective cohort study. | 2018 | NR | Prospective cohort | Primary report | 524 | 524 | 20 | 11 | Moderate |
| [23] | S03 | Richter 2019 | Rheumatic Syndromes Associated With Immune Checkpoint Inhibitors: A Single-Center Cohort of Sixty-One Patients. | 2019 | USA | Retrospective cohort | Primary report | 1293 | 1293 | 34 | 3 | High |
| [24] | S04 | López González 2023 | MULTIDISCIPLINARY PROSPECTIVE STUDY OF PATIENTS TREATED WITH IMMUNE CHECKPOINT INHIBITORS WHO DEVELOPED RHEUMATIC IMMUNERELATED ADVERSE EVENTS | 2023 | NR | Prospective cohort | Conference abstract | NR | NR | NR | NR | High |
| [25] | S05 | Tran 2024 | Rapid identification of inflammatory arthritis and associated adverse events following immune checkpoint therapy: a machine learning approach. | 2024 | United States | Retrospective cohort; EHR | Primary report | 2451 | 2451 | 89 | NR | Moderate |
| [26] | S06 | Daoussis 2020 | An MRI study of immune checkpoint inhibitor-induced musculoskeletal manifestations myofasciitis is the prominent imaging finding. | 2020 | NR | Prospective cohort | Primary report | NR | NR | NR | NR | High |
| [27] | S07 | Llobell-Uriel 2025 | POS0859 POLYMYALGIA-LIKE SYNDROME IN PATIENTS TREATED WITH CHECKPOINT INHIBITORS: A FASTER PREDNISONE TAPERING REGIMEN AS AN EFFECTIVE TREATMENT APPROACH | 2025 | Spain | Retrospective cohort | Conference abstract | NR | NR | NR | 28 | High |
| [29] | S08 | Zimmer 2016 | Neurological, respiratory, musculoskeletal, cardiac and ocular side-effects of anti-PD-1 therapy | 2016 | NR | Retrospective multicentre cohort | Primary report | NR | NR | NR | NR | Moderate |
| [30] | S09 | Chan 2020 | Higher Checkpoint Inhibitor Arthritis Disease Activity may be Associated With Cancer Progression: Results From an Observational Registry. | 2020 | USA | Prospective registry study | Primary report | 66 | NR | 30 | 5 | Moderate |
| [35] | S10 | Gómez-Puerta 2022 | Clinical Patterns and Follow-Up of Inflammatory Arthritis and Other Immune-Related Adverse Events Induced by Checkpoint Inhibitors. A Multicenter Study. | 2022 | Spain | Retrospective multicentre case series | Primary report | 73 | NR | NR | NR | Moderate |
| [36] | S11 | Yoon 2025 | Comparative study of management strategies for immune checkpoint inhibitor-induced inflammatory arthritis: rheumatologists versus oncologists. | 2025 | Australia | Retrospective cohort | Primary report | 871 | 871 | 20 | NR | Moderate |
| [37] | S12 | Ladouceur 2023 | Immune checkpoint inhibitor rechallenge in patients who previously experienced immune-related inflammatory arthritis: a multicentre observational study. | 2023 | France | Retrospective cohort | Primary report | 23 | NR | 0 | 0 | High |
| [38] | S13 | Gente 2023 | Sex and anti-inflammatory treatment affect outcome of melanoma and non-small cell lung cancer patients with rheumatic immune-related adverse events. | 2023 | NR | Prospective cohort | Primary report | 91 | NR | NR | NR | Moderate |
| [39] | S14 | Lemaire 2025 | Neoadjuvant immunotherapy for nonmetastatic dMMR/MSI colon cancer: a real-world retrospective AGEO study. | 2025 | France | Retrospective cohort | Primary report | 40 | 32 | NR | NR | High |
| [40] | S15 | Chen 2020 | Immunotherapy as second-line treatment and beyond for non-small cell lung cancer in a single center of China: Outcomes, toxicities, and clinical predictive factors from a real-world retrospective analysis | 2020 | China | Retrospective cohort | Primary report | 2430 | 97 | NR | NR | Moderate |
| [41] | S16 | Bair 2019 | Outcomes and Toxicities of Programmed Death-1 (PD-1) Inhibitors in Hodgkin Lymphoma Patients in the United States: A Real-World, Multicenter Retrospective Analysis | 2018 | United States | Retrospective cohort | Primary report | 53 | 53 | NR | NR | High |
| [42] | S17 | Harnden 2025 | Whole-body MRI in patients with arthralgia or inflammatory arthritis after exposure to immune checkpoint inhibitors: a single-centre prospective imaging study. | 2025 | NR | Prospective cohort | Primary report | 60 | NR | 25 | 5 | Moderate |
| [43] | S18 | Di Matteo 2025 | Ultrasound-detected inflammation and structural changes in the joints, tendons and entheses of patients with cancer who developed arthritis or arthralgia after exposure to immune checkpoint inhibitors. | 2025 | NR | Retrospective cross-sectional case series | Primary report | 101 | NR | 48 | 5 | High |
| [44] | S19 | Ponce 2022 | Imaging Findings in Patients with Immune Checkpoint Inhibitor-Induced Arthritis. | 2022 | Spain | Retrospective observational case series | Primary report | 19 | NR | 14 | 5 | Moderate |
| [45] | S20 | Paiola 2025 | Osteoarthritis increases the risk of inflammatory arthritis due to immune checkpoint inhibitors associated with tissue-resident memory T cells. | 2025 | NR | Case-control study | Primary report | NR | NR | NR | NR | High |
| [46] | S21 | Gatto 2023 | Early Increase of Circulating Transitional B Cells and Autoantibodies to Joint-Related Proteins in Patients With Metastatic Melanoma Developing Checkpoint Inhibitor-Induced Inflammatory Arthritis. | 2022 | Sweden | Retrospective analysis of prospectively collected data | Primary report | 22 | NR | 7 | 1 | Moderate |
| [47] | S22 | Cappelli 2023 | TWO DISTINCT SUBSETS OF IMMUNE CHECKPOINT INHIBITOR-INDUCED INFLAMMATORY ARTHRITIS DEFINED BY LATENT CLASS ANALYSIS | 2023 | United States of America | Retrospective cohort | Conference abstract | 126 | NR | 126 | NR | Moderate |
| [48] | S23 | Lidar 2018 | Rheumatic manifestations among cancer patients treated with immune checkpoint inhibitors. | 2018 | Israel | Retrospective cohort | Primary report | 400 | 400 | 9 | 0 | Moderate |
| [49] | S24 | Cappelli 2017 | Inflammatory arthritis and sicca syndrome induced by nivolumab and ipilimumab. | 2017 | USA | Retrospective case series | Primary report | 13 | NR | 9 | 0 | Moderate |
| [50] | S25 | Smith 2019 | Arthritis After Cancer Immunotherapy: Symptom Duration and Treatment Response. | 2019 | USA | Retrospective case series | Primary report | 10 | NR | 8 | 1 | Moderate |
| [51] | S26 | Ladouceur 2025 | Chronicity of Immune Checkpoint Inhibitor-Associated Inflammatory Arthritis After Immunotherapy Discontinuation: Results From the Canadian Research Group of Rheumatology in Immuno-Oncology Database. | NR | Canada | Retrospective cohort | Primary report | 367 | 367 | 172 | NR | Moderate |
| [52] | S27 | Goodman 2023 | Extended Follow-Up of Chronic Immune-Related Adverse Events Following Adjuvant Anti-PD-1 Therapy for High-Risk Resected Melanoma. | 2023 | US and Australia | Retrospective cohort | Follow-up report | 318 | 318 | NR | NR | High |
| [53] | S28 | Patrinely 2021 | Chronic Immune-Related Adverse Events Following Adjuvant Anti-PD-1 Therapy for High-risk Resected Melanoma. | 2021 | United States; Australia | Retrospective cohort | Primary report | 387 | 387 | NR | NR | High |
| [54] | S29 | Ladouceur 2025 | Resolution of immune checkpoint inhibitors-induced inflammatory arthritis while maintaining active treatment with checkpoint inhibitors and after its discontinuation: An observational study. | 2025 | France | Retrospective observational study | Primary report | 80 | NR | 80 | NR | High |
| [55] | S30 | Liapi 2022 | TREATMENT of IMMUNE CHECKPOINT INHIBITOR-INDUCED INFLAMMATORY ARTHRITIS and POLYMYALGIA RHEUMATICA | 2022 | Sweden | Retrospective case series | Conference abstract | 20 | NR | 14 | 9 | Moderate |
| [56] | S31 | Hysa 2025 | Clinical outcomes in cancer patients with immune checkpoint inhibitor-induced arthritis treated with methotrexate: a retrospective longitudinal monocentric pilot study. | 2025 | Italy | Retrospective cohort | Primary report | 14 | NR | 14 | 0 | Moderate |
| [57] | S32 | Roberts 2019 | Hydroxychloroquine is a safe and effective steroid-sparing agent for immune checkpoint inhibitor-induced inflammatory arthritis. | 2019 | Canada | Retrospective case series | Primary report | 11 | NR | 11 | 1 | Moderate |
| [58] | S33 | Alperin 2017 | Traditional disease modifying anti-rheumatic drugs (tDMARDs), hydroxychloroquine (HCQ) and/or sulfasalazine (SSZ), are rapidly effective in immune checkpoint inhibitors-induced inflammatory arthritis | 2017 | USA | Retrospective case series | Conference abstract | 6 | NR | 6 | 0 | Moderate |
| [59] | S34 | Petit 2025 | Tocilizumab provides dual benefits in treating immune checkpoint inhibitor-associated arthritis and preventing relapse during ICI rechallenge: the TAPIR study. | 2024 | Switzerland | Retrospective cohort | Primary report | 31 | NR | 26 | 7 | High |
| [61] | S35 | Leipe 2018 | Characteristics and treatment of new-onset arthritis after checkpoint inhibitor therapy. | 2018 | Germany | Prospective cohort; rheumatology referral cohort | Primary report | 16 | NR | 14 | 5 | High |
| [62] | S36 | Tison 2026 | Comparing immune-checkpoint inhibitor-mediated inflammatory arthritis to polymyalgia rheumatica: a prospective cohort study. | 2026 | USA | Prospective cohort; registry study | Primary report | 490 | NR | 418 | 72 | Moderate |
| [63] | S37 | Bass 2025 | Incidence of checkpoint inhibitor-associated inflammatory arthritis, immunomodulation and mortality in cancer patients on immunotherapy: a retrospective cohort study. | 2024 | USA | Retrospective cohort | Primary report | 3119 | 3119 | 153 | NR | High |
| [64] | S38 | Kobashigawa 2025 | Rheumatic Immune-Related Adverse Events: Immune Checkpoint Inhibitor Therapy and Ramifications for Health Disparities. | 2025 | USA | Retrospective cohort | Primary report | 611 | 611 | 14 | NR | High |
| [65] | S39 | Barasch 2025 | Checkpoint Inhibitor Inflammatory Arthritis: Single Center Case Identification and Chart Validation | 2025 | USA | Retrospective cohort; EHR; claims | Conference abstract | 500 | 500 | 44 [HSS Chart Review]; Not specified separately [Komodo] | 7 [HSS Chart Review] | Moderate |
| [66] | S40 | Cunningham-Bussel 2022 | Predictors of Rheumatic Immune-Related Adverse Events and De Novo Inflammatory Arthritis After Immune Checkpoint Inhibitor Treatment for Cancer. | 2021 | NR | Retrospective cohort; EHR | Primary report | 8028 | 8028 | 118 | NR | High |
| [67] | S41 | Kaur 2019 | Immune-related adverse events in cancer patients treated with immune checkpoint inhibitors: A single-center experience | 2019 | USA | Retrospective cohort | Primary report | 220 | 220 | 2 | 0 | Moderate |
| [68] | S42 | Moussa 2026 | Efficacy of Immune Checkpoint Blockade in Advanced Upper Tract Urothelial Cancer With DNA Mismatch Repair Deficiency or Microsatellite Instability. | 2026 | USA | Retrospective cohort | Primary report | 119 | 24 | NR | NR | High |
| [69] | S43 | Li 2025 | Effect of Age on Rheumatic Immune-Related Adverse Events: Experience From the Canadian Research Group of Rheumatology in Immuno-Oncology (CanRIO). | 2024 | Canada | Prospective cohort | Primary report | 139 | NR | 117 | NR | Moderate |
| [70] | S44 | Dagogo-Jack 2025 | Durvalumab Combined With Pemetrexed-Based Chemotherapy in Trial-Ineligible Patients With Mesothelioma: A Brief Report. | 2025 | USA | Retrospective case series | Primary report | 12 | 12 | NR | NR | Moderate |
| [71] | S45 | Sabatier 2018 | Nivolumab in routine practice for older patients with advanced or metastatic non-small cell lung cancer | 2018 | France | Retrospective cohort | Primary report | 30 | 30 | NR | NR | High |
| [72] | S46 | Pozas 2025 | Late-Onset Immune-Related Adverse Events in Patients with Advanced Melanoma: The LATENT Study. | 2025 | UK | Retrospective cohort | Primary report | 246 | 246 | NR | NR | Moderate |
| [73] | S47 | Kaushik 2025 | Real world experience on patterns of usage and toxicity profile of immunotherapy drugs in Indian patients: A prospective observational study | 2023 | India | Prospective cohort | Primary report | 53 | 53 | NR | NR | High |
| [74] | S48 | Kitano 2024 | Delayed immune-related adverse events in long-responders of immunotherapy: a single-center experience. | 2024 | Japan | Retrospective cohort | Primary report | 336 | 336 | 5 | NR | High |
| [75] | S49 | Azuma 2022 | Utility of periodic medical questionnaires and examinations for immune-related adverse event screening: A prospective observational study | 2022 | Japan | Prospective cohort | Primary report | 51 | 51 | 1 | 0 | High |
| [76] | S50 | Yeung 2022 | No association between BMI and immunotoxicity or clinical outcomes for immune checkpoint inhibitors | 2022 | Canada | Retrospective cohort | Primary report | 409 | 409 | NR | NR | Moderate |
| [77] | S51 | Salvatierra Velasco 2022 | PREVALENCE of IMMUNORELATED MUSCULOSKELETAL ADVERSE EFFECTS in PATIENTS with LUNG CANCER or MELANOMA TREATED with IMMUNO-CHECKPOINT INHIBITORS | NR | NR | NR | Primary report | NR | NR | NR | NR | High |
| [79] | S52 | Ahern 2021 | Retrospective analysis of hospital admissions due to immune checkpoint inhibitor-induced immune-related adverse events (irAE) | 2020 | Australia | Retrospective cohort | Primary report | 140 | 140 | NR | NR | High |
| [80] | S53 | Fukui 2019 | Activity of Nivolumab and Utility of Neutrophil-to-Lymphocyte Ratio as a Predictive Biomarker for Advanced Non-Small-Cell Lung Cancer: A Prospective Observational Study. | 2018 | Japan | Prospective cohort | Primary report | 52 | 52 | 1 | 0 | Moderate |
| [81] | S54 | Le Burel 2017 | Prevalence of immune-related systemic adverse events in patients treated with anti-Programmed cell Death 1/anti-Programmed cell Death-Ligand 1 agents: A single-centre pharmacovigilance database analysis. | 2017 | France | Retrospective pharmacovigilance database analysis | Primary report | 908 | 908 | 14 (detailed series); 19 (prevalence table sum) | 4 (detailed series); 2 (prevalence table) | High |
| [82] | S55 | Fitzpatrick 2026 | Evaluating Hip Osteoarthritis as a Risk Factor for Immune Checkpoint Inhibitor-Induced Inflammatory Arthritis. | NR | USA | Retrospective cohort | Primary report | 309 | 309 | 42 | NR | Moderate |
| [83] | S56 | Ceccarelli 2023 | Rheumatic Diseases Development in Patients Treated by Anti-PD1 Immune Checkpoint Inhibitors: A Single-Centre Descriptive Study. | 2023 | Italy | Retrospective descriptive case series | Primary report | 32 | NR | 20 | 6 | Moderate |
| [84] | S57 | Liapi 2023 | RISK FACTORS FOR DEVELOPING RHEUMATIC IMMUNE-RELATED ADVERSE EVENTS AFTER IMMUNOTHERAPY WITH IMMUNE CHECKPOINT INHIBITORS | 2023 | Sweden | Retrospective cohort | Conference abstract | 118 | 118 | 6 | 3 | Moderate |
| [85] | S58 | Bilbao 2019 | Rheumatological immune-related adverse events associadted with immunotherapy in solid organ tumor. Study of 102 cases from a referral single center for last 4 years | 2019 | Spain | Retrospective cohort/case series | Conference abstract | 102 | NR | NR | NR | High |
| [86] | S59 | León 2019 | Adverse autoimmune events in cancer patientstreated with immunotherapy. Analysis of cases between 2011 and 2018 | 2019 | Spain | Retrospective cohort | Conference abstract | 190 | 190 | NR | NR | Moderate |
| [87] | S60 | Cretu 2022 | Rheumatological Adverse Events Following Immunotherapy for Cancer. | 2022 | Romania | Retrospective cohort study | Primary report | 348 | 348 | NR | NR | High |
| [88] | S61 | Ladouceur 2023 | Resolution of Immune Checkpoint Inhibitors-Induced Inflammatory Arthritis While Maintaining Active Treatment with Checkpoint Inhibitors | 2023 | France | Single-centre retrospective rheumatology referral case series | Conference abstract | 80 | NR | 80 | NR | High |
| [89] | S62 | Thimmapuram 2023 | Identification of Biomarkers Predicting Steroid Resistance in Immune Checkpoint Inhibitor-Induced Arthritis | 2023 | USA | Prospective cohort | Conference abstract | 39 | NR | 39 | NR | High |
| [90] | S63 | Bruce 2022 | PREDICTORS of RHEUMATIC TOXICITIES of IMMUNE CHECKPOINT INHIBITORS and CANCER OUTCOMES in PATIENTS with ADVANCED MELANOMA | 2022 | NR | NR | Conference abstract | NR | NR | NR | NR | High |
| [91] | S65 | Liew 2019 | Association of good oncological response to therapy with the development of rheumatic immune-related adverse events following PD-1 inhibitor therapy. | 2019 | Australia | Retrospective cohort | Primary report | 244 | 244 | 11 | 1 | High |
| [92] | S65 | Melia 2023 | Musculoskeletal immune-related adverse events in 927 patients treated with immune checkpoint inhibitors for solid cancer. | 2023 | France | Retrospective cohort | Primary report | 927 | 927 | NR | NR | Moderate |

Occurrence-analysis note: Supplementary Table S1 preserves author-reported phenotype counts from the source reports. These values are not necessarily identical to the phenotype-specific numerator used in the denominator-valid occurrence meta-analysis when phenotype separation or source-cohort mapping was required. The study-level occurrence assessment is provided in Supplementary Table S5. A value of 0 in this descriptive table must not be treated as a confirmed occurrence-analysis zero unless separate outcome assessment and denominator validity have been established. The Melia 2023 full report is reference [92]; its earlier conference abstract is retained as reference [78] and is not an additional row. The Ladouceur 2023 conference abstract (reference [88]) and 2025 full report (reference [54]) remain linked to the same study family. The 2026 Llobell-Uriel cohort [28] is retained as contextual clinical-course evidence but is not included in the primary de novo PMR pool because case-level de novo timing and possible overlap with the 2025 POS0859 report [27] could not be verified from the available report-level record; this decision was not based solely on other autoimmune conditions.

# **Supplementary Table S2. Sensitivity analyses**

*ICI-IA estimates were numerically stable; ICI-PMR estimates were directionally consistent but sparse and unstable. A prediction interval was not estimable for k = 2.*

| **Sensitivity analysis** | **Parent outcome** | **k** | **Events** | **Total** | **Pooled proportion (%)** | **95% CI low (%)** | **95% CI high (%)** | **95% PI low (%)** | **95% PI high (%)** | **I² (%)** | **tau²** | **Relative change vs main (%)** | **Interpretation** |
| --- | --- | --- | --- | --- | --- | --- | --- | --- | --- | --- | --- | --- | --- |
| ICI-IA: exclude conference abstracts | De-novo ICI-IA occurrence | 4 | 133 | 4,463 | 2.76 | 1.85 | 4.09 | 0.79 | 9.15 | 78.4 | 0.115 | -4.0 | Numerically stable |
| ICI-IA: include lower-detail source cohorts | De-novo ICI-IA occurrence | 6 | 143 | 4,774 | 2.82 | 2.04 | 3.90 | 1.19 | 6.53 | 65.4 | 0.088 | -1.8 | Numerically stable |
| ICI-IA: strict Buder imaging definition | De-novo ICI-IA occurrence | 5 | 137 | 4,644 | 2.72 | 1.97 | 3.73 | 1.16 | 6.23 | 66.0 | 0.070 | -5.5 | Numerically stable |
| ICI-PMR: exclude conference abstracts | De-novo ICI-PMR/PMR-like occurrence | 2 | 14 | 1,817 | 0.71 | 0.15 | 3.36 | NR | NR | 91.3 | 1.098 | -34.3 | Direction consistent; magnitude unstable and sparse |
| ICI-PMR: include lower-detail source cohorts | De-novo ICI-PMR/PMR-like occurrence | 4 | 21 | 2,494 | 0.85 | 0.29 | 2.44 | 0.03 | 22.61 | 82.5 | 0.930 | -21.3 | Direction consistent; magnitude unstable and sparse |
| ICI-IA: exclude Tran cohort | De-novo ICI-IA occurrence | 4 | 51 | 2,193 | 2.61 | 1.50 | 4.79 | 1.09 | 6.13 | 78.0 | NR | -9.1 | Leave-one-cohort-out sensitivity; pooled estimate remained similar |

Sensitivity analyses included exclusion of the Tran cohort, exclusion of the conference abstract, addition of a lower-detail source cohort, and use of the stricter imaging-supported numerator.

# Supplementary Table S3. GRADE summary of findings for pooled estimands

*Certainty ratings apply to the four pooled outcomes. The table summarizes risk of bias, inconsistency, indirectness, imprecision, and publication-bias judgments; these are outcome-level assessments rather than additional study-level observations.*

| **Outcome** | **k** | **Pooled proportion (%)** | **95% CI** | **95% prediction interval** | **Events / total** | **Risk-of-bias distribution** | **Starting certainty** | **Downgrade summary** | **Final certainty** | **Key rationale** |
| --- | --- | --- | --- | --- | --- | --- | --- | --- | --- | --- |
| Conventional synthetic DMARD use | 2 | 32.52 | 10.201 to 67.149 | Not estimable | 22 / 64 | High: 1; Moderate: 1 | Low | RoB: Serious; Inconsistency: Serious; Indirectness: Serious; Imprecision: Serious; Publication bias: Undetected | Very low | Very low certainty because selected observational case cohorts, serious risk of bias, substantial unexplained heterogeneity, indirectness, and a very wide confidence interval leave the true utilisation proportion highly uncertain. |
| De-novo ICI-IA crude occurrence proportion | 5 | 2.87 | 2.021 to 4.073 | 1.064 to 7.531 | 140 / 4644 | High: 3; Moderate: 2 | High | RoB: Serious; Inconsistency: Serious; Indirectness: Not serious; Imprecision: Not serious; Publication bias: Undetected | Low | Low certainty after one-level downgrades for serious risk of bias and serious inconsistency; the pooled estimate is informative but may differ materially across clinical settings. |
| De-novo ICI-PMR/PMR-like crude occurrence proportion | 3 | 1.08 | 0.316 to 3.622 | 0.007 to 63.425 | 19 / 1998 | High: 2; Moderate: 1 | High | RoB: Serious; Inconsistency: Serious; Indirectness: Not serious; Imprecision: Very serious; Publication bias: Undetected | Very low | Very low certainty because serious risk of bias and inconsistency combine with very serious imprecision; the pooled percentage should be treated as a provisional range rather than a stable population frequency. |
| Systemic glucocorticoid use | 2 | 61.74 | 40.196 to 79.482 | Not estimable | 69 / 123 | High: 1; Moderate: 1 | Low | RoB: Serious; Inconsistency: Serious; Indirectness: Serious; Imprecision: Serious; Publication bias: Undetected | Very low | Very low certainty because selected observational case cohorts, serious risk of bias, substantial unexplained heterogeneity, indirectness, and a wide confidence interval make the true utilisation proportion highly uncertain. |

# Supplementary Table S4. Structured synthesis without meta-analysis

*Counts are theme-specific routing units and are not additive counts of independent studies. In total, 61 reports representing 59 independent studies contributed to at least one SWiM theme.*

| **Clinical theme** | **Studies** | **Evidence groups** | **Source rows** | **Direction** | **Reason not pooled** |
| --- | --- | --- | --- | --- | --- |
| Occurrence and measurement | 32 | 23 | 37 | Consistent occurrence; magnitude varied by measurement frame | Incompatible denominators, ascertainment, event definitions, and time scales |
| Clinical phenotype and patient burden | 4 | 5 | 5 | Heterogeneous phenotypes; no common burden scale | Different phenotype definitions and mostly descriptive measures |
| Persistence, remission, and relapse | 11 | 15 | 15 | Persistence and remission coexisted | Different time origins, follow-up horizons, definitions, and treatment exposures |
| Systemic glucocorticoid treatment | 11 | 13 | 13 | Frequently used, with context-dependent variation | Ever-use, current-use, and fixed-time measures were not comparable |
| DMARD escalation and treatment response | 17 | 19 | 20 | Escalation and response signals occurred but were uncontrolled | Drug classes, indications, response definitions, and denominators differed |
| Management of ICI therapy | 7 | 7 | 7 | Holding or stopping was common in some settings but not universal | Interruption, discontinuation, continuation, and rechallenge were distinct actions |
| Oncologic outcomes | 4 | 4 | 4 | Insufficient for an IA/PMR-attributable oncologic effect | Cancer types, endpoints, timing, and comparators were incompatible |

**Supplementary Table S5. Study-level assessment of denominator-valid occurrence evidence for ICI-associated inflammatory arthritis and PMR/PMR-like syndromes**

| **Study** | **ICI cohort n** | **IA numerator used** | **PMR numerator / status** | **Source-reported phenotype count / context** | **De novo / flare assessment** | **Anti-CTLA-4 combination proportion** | **Age distribution** | **PMR age ≥50 numerator / denominator** | **Occurrence inclusion rationale** |
| --- | --- | --- | --- | --- | --- | --- | --- | --- | --- |
| Buder-Bakhaya 2018 [21] | 195 | 10 | NR | NR | Exclusion of pre-existing rheumatic disease was not fully detailed; retained with Tier B definition. | 36/195 (18.5%) reported | Median 63.9 years (range 17-91) | NR | Denominator-valid IA cohort |
| Kostine 2018 [22] | 524 | 9 | 11 | 20 inflammatory rheumatic presentations (7 RA-like, 11 PMR-like, 2 PsA-like) | Phenotype-specific source data separated 9 non-PMR IA cases from 11 PMR-like cases; no additional flare count was inferred. | NR | Mean 64.5 years (SD 12.8) | NR | Denominator-valid IA and PMR cohorts; PMR-like cases analyzed separately from IA. |
| Richter 2019 [23] | 1,293 | 25 | 3 | 34 IA cases in the overall 61-patient rheumatology series | Three new-onset PMR-like cases retained; one pre-existing PMR flare excluded. The overall rheumatology series included patients outside the denominator-defined source cohort. | NR | Mean 62.6/61.3 years (phenotype-specific reporting) | NR | 25 IA cases mapped to the denominator-valid 1,293-patient source cohort. |
| López González 2023 [24] | 181 | 7 | 5 | 21 rheumatic ir-AEs | Limited conference-abstract case-level detail; retained for primary analysis with publication-type sensitivity. | NR | NR | NR | Denominator-valid conference-abstract cohort |
| Tran 2024 [25] | 2,451 | 89 | NR | 89 | Chart-adjudicated de novo ICI-associated cases; broad clinician-suspected definition; retained in primary analysis and excluded in sensitivity analysis. | NR | NR | NR | Denominator-valid broad-definition cohort |
| Daoussis 2020 [26] | 130 | 3 | NR | 3 IA events used in lower-detail sensitivity analysis | Source detail was insufficient for the primary occurrence pool; no additional flare status was inferred. | NR | NR | NR | Included only in the lower-detail ICI-IA sensitivity analysis (3/130). |
| Llobell-Uriel 2026 [28] | 734 | NR | 12 reported; not used | NR | The report described 12 PMR cases, but the available report-level information did not permit case-level verification of de novo timing or separation from pre-existing disease; possible overlap with the 2025 POS0859 report could not be independently confirmed. Retained as contextual evidence rather than pooled. | NR | NR | NR | Contextual clinical-course evidence; not included in the primary de novo PMR pool because case-level de novo status and possible overlap with POS0859 could not be verified from the available report-level record. The decision was not based solely on other autoimmune conditions; no pooled estimate was changed. |
| Zimmer 2016 [29] | 496 | NR | 2 | 2 PMR/PMR-like events used in lower-detail sensitivity analysis | Source detail was insufficient for the primary PMR pool; no additional flare status was inferred. | NR | NR | NR | Included only in the lower-detail PMR sensitivity analysis (2/496). |

*Abbreviations: IA, inflammatory arthritis; ICI, immune checkpoint inhibitor; PMR, polymyalgia rheumatica; NR, not reported or not derivable. NR outcomes were not coded as zero events. No additional confirmed denominator-valid zero-event PMR cohort was identified. Age-specific PMR numerators and ICI-treated denominators were not consistently available, so an age-restricted pooled estimate was not calculated. The 2026 Llobell-Uriel report [28] described 12 PMR cases among 734 ICI-treated patients and was retained as contextual evidence rather than added to the primary de novo PMR pool because case-level de novo timing and possible overlap with the 2025 POS0859 report [27] could not be verified from the available report-level record. The exclusion was not based solely on the presence of other autoimmune conditions; no pooled estimate was changed.*

**Supplementary Table S6. Source data for the contextual melanoma-trial CTCAE arthralgia analysis**

| **Trial** | **ICI arm, n** | **All-grade CTCAE arthralgia, n** | **Observed proportion** | **ICI-containing regimen / analysis role** |
| --- | --- | --- | --- | --- |
| Ascierto 2023 IMspire150 [31] | 231 | 103 | 44.59% | Atezolizumab + vemurafenib + cobimetinib |
| Dummer 2022 COMBI-i [32] | 267 | 62 | 23.22% | Spartalizumab + dabrafenib + trametinib |
| Eggermont 2018 KEYNOTE-054 [33] | 509 | 64 | 12.57% | Adjuvant pembrolizumab |
| Long 2022 KEYNOTE-716 [34] | 483 | 115 | 23.81% | Adjuvant pembrolizumab |
| Total / pooled | 1,490 | 344 | GLMM pooled 24.57% (95% CI 11.85%-43.47%) | Kept separate from ICI-IA; 95% PI 7.05%-58.33%; I²=64.2% |

*CTCAE, Common Terminology Criteria for Adverse Events; CI, confidence interval; GLMM, generalized linear mixed model; ICI-IA, immune checkpoint inhibitor-associated inflammatory arthritis; PI, prediction interval. These symptom-level arthralgia data were analyzed separately and were not pooled with ICI-IA. Event counts and safety denominators were extracted from Roberts et al., Table 2 [10]. Trial identities are given below; IMspire150 and COMBI-i included background BRAF/MEK targeted treatment. The four trials provide contextual evidence and do not increase the 65-report core review count.*

# Supplementary Figure Legends

**Supplementary Figure S1. PMR/PMR-like syndrome after excluding the conference abstract. Sensitivity analysis.**

**Supplementary Figure S2. PMR/PMR-like syndrome including the lower-detail source cohort. Sensitivity analysis.**

**Supplementary Figure S3. Conventional synthetic DMARD use among confirmed ICI-IA cases. Study-specific proportions and the exploratory random-effects estimate are shown with 95% confidence intervals.**

**Supplementary Figure S4. Systemic glucocorticoid use among confirmed ICI-IA cases. Study-specific proportions and the exploratory random-effects estimate are shown with 95% confidence intervals.**
